# Synthesis and Biological Evaluation of *N*-Alkoxyphenyl-3-hydroxynaphthalene-2-carboxanilides

**DOI:** 10.3390/molecules20069767

**Published:** 2015-05-27

**Authors:** Tomas Gonec, Iveta Zadrazilova, Eoghan Nevin, Tereza Kauerova, Matus Pesko, Jiri Kos, Michal Oravec, Peter Kollar, Aidan Coffey, Jim O’Mahony, Alois Cizek, Katarina Kralova, Josef Jampilek

**Affiliations:** 1 Department of Chemical Drugs, Faculty of Pharmacy, University of Veterinary and Pharmaceutical Sciences, Palackeho 1/3, 61242 Brno, Czech Republic; E-Mails: zadrazilova.iveta@seznam.cz (I.Z.); jurd@email.cz (J.K.); 2 Department of Infectious Diseases and Microbiology, Faculty of Veterinary Medicine, University of Veterinary and Pharmaceutical Sciences, Palackeho 1/3, 61242 Brno, Czech Republic; E-Mail: cizeka@vfu.cz; 3 Department of Biological Sciences, Cork Institute of Technology, Bishopstown, Cork, Ireland; E-Mails: eoghan.nevin@mycit.ie (E.N.); aidan.coffey@cit.ie (A.C.); jim.omahony@cit.ie (J.O.); 4 Department of Human Pharmacology and Toxicology, Faculty of Pharmacy, University of Veterinary and Pharmaceutical Sciences, Palackeho 1/3, 61242 Brno, Czech Republic; E-Mails: tereza.kauerova@gmail.com (T.K.); kollarp@vfu.cz (P.K.); 5 Department of Environmental Ecology, Faculty of Natural Sciences, Comenius University, Mlynska dolina Ch-2, 84215 Bratislava, Slovakia; E-Mail: matus.pesko@gmail.com; 6 Global Change Research Centre AS CR, Belidla 986/4a, 60300 Brno, Czech Republic; E-Mail: oravec.m@czechglobe.cz; 7 Institute of Chemistry, Faculty of Natural Sciences, Comenius University, Mlynska dolina Ch-2, 84215 Bratislava, Slovakia; E-Mail: kata.kralova@gmail.com

**Keywords:** hydroxynaphthalene-2-carboxanilides, *in vitro* antibacterial activity, *in vitro* antimycobacterial activity, *in vitro* cytotoxicity, photosynthetic electron transport inhibition, structure-activity relationships

## Abstract

A series of fifteen new *N*-alkoxyphenylanilides of 3-hydroxynaphthalene-2-carboxylic acid was prepared and characterized. Primary *in vitro* screening of the synthesized compounds was performed against *Staphylococcus aureus*, three methicillin-resistant *S. aureus* strains, *Mycobacterium tuberculosis* H37Ra and *M. avium* subsp. *paratuberculosis.* Some of the tested compounds showed antibacterial and antimycobacterial activity against the tested strains comparable with or higher than that of the standards ampicillin or rifampicin. 3-Hydroxy-*N*-(2-propoxyphenyl)naphthalene-2-carboxamide and *N*-[2-(but-2-yloxy)-phenyl]-3-hydroxynaphthalene-2-carboxamide had MIC = 12 µM against all methicillin-resistant *S. aureus* strains; thus their activity is 4-fold higher than that of ampicillin. The second mentioned compound as well as 3-hydroxy-*N*-[3-(prop-2-yloxy)phenyl]-naphthalene-2-carboxamide had MICs = 23 µM and 24 µM against *M. tuberculosis* respectively. *N*-[2-(But-2-yloxy)phenyl]-3-hydroxynaphthalene-2-carboxamide demonstrated higher activity against *M. avium* subsp. *paratuberculosis* than rifampicin. Screening of the cytotoxicity of the most effective antimycobacterial compounds was performed using THP-1 cells, and no significant lethal effect was observed for the most potent compounds. The compounds were additionally tested for their activity related to inhibition of photosynthetic electron transport (PET) in spinach (*Spinacia oleracea* L.) chloroplasts. *N*-(3-Ethoxyphenyl)-3-hydroxynaphthalene-2-carboxamide (IC_50_ = 4.5 µM) was the most active PET inhibitor. The structure-activity relationships are discussed.

## 1. Introduction

Recent studies have shown that, despite antibacterial therapy, methicillin-resistant *Staphylococcus aureus* (MRSA) infections are still associated with serious clinical consequences, especially treatment failure, higher morbidity and mortality [1], prolonged hospitalization [2], increased health care costs [2], *etc.* Activity against MRSA is of a great importance in the new generation of antibacterial agents because of the worldwide increasing prevalence of this pathogen [3], more frequent antibiotic resistance to available anti-MRSA drugs, their toxicity and general lack of oral agents [4]. Tuberculosis (TB), caused by *Mycobacterium tuberculosis* and its multidrug-resistant (MDR) or extensively drug-resistant (XDR) strains also remains one of the world’s deadliest communicable diseases. In 2013, an estimated 9.0 million people developed TB and 1.5 million died from the disease. Drug resistance surveillance data indicate that in 2013 approximately 480,000 people developed MDR-TB worldwide. XDR-TB has been identified in 100 countries globally as of 2013, and the average proportion of MDR-TB cases with XDR-TB was 9.0% [5]. Because of *M. tuberculosis*, the pathogenic role of non-tuberculous mycobacteria (NTM) in humans was overshadowed for a long time. NTM have become more prevalent human pathogens, causing difficult-to-treat or incurable diseases, potentially ending in death when the patient is immunocompromised. NTM can cause a broad spectrum of diseases, such as pulmonary disease, lymphadenitis, skin and soft tissue disease, gastrointestinal and skeletal infections. They are resistant to standard antimycobacterial therapy, but may be susceptible to some standard antibiotics. However, the resistance to these antibiotics develops quickly [6].

3-Hydroxynaphthalene-2-carboxamides can be considered as cyclic analogues of salicylanilides that have expressed promising results as potential antimicrobial and antimycobacterial agents ([7,8] and refs. therein). The anti-infectious effect is connected with the ability of salicylanilides to inhibit various enzymatic systems in bacteria, e.g., two-component regulatory systems (TCS), transglycosylases from *Staphylococcus aureus* (but not from *M. tuberculosis*), D-alanine-d-alanine ligase, isocitrate lyase and methionine aminopeptidase ([7,8] and refs. therein). They serve also as inhibitors of protein kinase epidermal growth factor receptor and are generally designed to compete with ATP for binding in catalytic domain of tyrosin kinase [9] or as selective inhibitors of interleukin-12p40 production [10].

Since 3-hydroxy-*N*-(methoxyphenyl)naphthalene-2-carboxamide showed activity against *Staphylococcus* strains and against mycobacterial species [11], other alkoxy derivatives were designed as homologues of those methoxyphenyl derivatives. The present work is focused on synthesis and investigation of the biological activity of *N*-alkoxyphenyl-3-hydroxynaphthalene-2-carboxanilides as promising antibacterial and antimycobacterial agents. Additionally all the compounds were tested for their ability to inhibit the photosynthetic electron transport (PET) in spinach (*Spinacia oleracea* L.) chloroplasts using the Hill reaction. This idea is based on the fact that both pharmaceuticals and pesticides are designed to target particular biological functions, and in some cases these functions overlap in their molecular target sites, or they target similar processes or molecules. Taking into the consideration that herbicides may also have molecular sites of action in mammals, until recently most pharmaceutical companies had pesticide divisions, and all compounds generated by either division of the company were evaluated for both pesticide and pharmaceutical uses. In the past, some leading pesticides have become pharmaceuticals and *vice versa* [12,13,14,15]. Moreover, a good correlation between microbiological activity and herbicidal effect was described in previous studies [16,17,18,19,20,21,22,23].

## 2. Results and Discussion

### 2.1. Chemistry

**Scheme 1:**
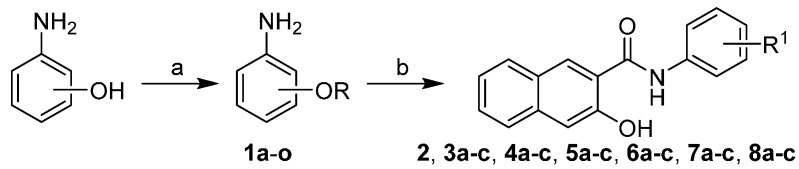
Synthesis of *N*-alkoxyphenyl-3-hydroxynaphthalene-2-carboxanilides.

The condensation of 3-hydroxynaphthalene-2-carboxylic acid with the appropriate alkoxy-substituted anilines using phosphorus trichloride in dry chlorobenzene under microwave conditions yielded a series of nineteen *N*-substituted 3-hydroxynaphthalene-2-carboxanilides **2**–**8c**. Unique commercially unavailable alkoxyanilines **1a**–**o** were prepared by a modified procedure according to De Marco *et al.* [24] using direct alkylation of corresponding aminophenols by alkylbromides in the presence of sodium hydride, see Scheme 1. Compounds **3a**–**8c** have not been described in literature so far. Compounds **2**–**3c** were published by Kos *et al.* [11], nevertheless they are also mentioned here to provide a complete overview of biological activities and structure-activity relationships.

The well-known physicochemical descriptors such as lipophilicity, surface tension of compounds, electronic parameters and molar volume of substituents are largely employed in structure-activity relationship analysis. In a number of studies examining biological activity of potential drugs, the relationship between lipophilicity or other descriptors and their potency have been investigated. In the current investigation the calculated lipophilicity (log *P*) and the surface tension of the compounds as well as the molar volume of R substituents (see Table 1), were used to determine if these factors play a role in their biological activity.

*Reagents and conditions*: (**a**) R-Br, NaH, acetonitrile, RT, 24 h; (**b**) 3-hydroxynaphthalene-2-carboxylic acid, PCl_3_, chlorobenzene, MW, 15 min.

**Table 1:** Structures of the discussed ring-substituted 3-hydroxynaphthalene-2-carboxanilides **2**–**8c**; calculated values of log *P*, surface tension (ST [dyne/cm]) and molar volume (MV [cm^3^]) of R substituents; *in vitro* antibacterial activity (MIC) of compounds in comparison with ampicillin (APC) standard; *in vitro* antimycobacterial activity (MIC) of compounds in comparison with rifampicin (RIF) standard; *in vitro* cytotoxicity assay (LD_50_) of chosen compounds; and IC_50_ values related to PET inhibition in spinach chloroplasts in comparison with 3-(3,4-dichlorophenyl)-1,1-dimethylurea (DCMU) standard.

Comp.	R^1^	log *P* *^a^*	MV *^a^* [cm^3^]	ST *^a^* [dyne/cm]	[µM]							
					MIC						LD_50_	PET IC_50_
					SA	MRSA 63718	MRSA SA 630	MRSA 3202	MT	MAP		
**2**	H	4.52	0	0	972	972	972	972	950	950	>30	ND
**3a**	2-OCH_3_	4.61	37.15	58.71	**55**	**55**	**55**	**55**	**51**	205	>30	59.5
**3b**	3-OCH_3_	4.56	37.15	58.71	873	873	873	873	**51**	426	>30	53.4
**3c**	4-OCH_3_	4.37	37.15	58.71	873	873	873	873	852	852	>30	ND
**4a**	2-OC_2_H_5_	4.92	53.66	56.66	832	832	832	832	813	813	>30	76.1
**4b**	3-OC_2_H_5_	4.88	53.66	56.66	832	832	832	832	813	813	>30	**4.5**
**4c**	4-OC_2_H_5_	4.67	53.66	56.66	832	832	832	832	813	813	>30	ND
**5a**	2-OC_3_H_7_	5.26	70.16	54.92	**12.4**	**12.4**	**12.4**	**12.4**	778	778	>30	128
**5b**	3-OC_3_H_7_	5.21	70.16	54.92	796	796	796	796	778	778	>30	**4.8**
**5c**	4-OC_3_H_7_	5.27	70.16	54.92	796	796	796	796	778	778	>30	ND
**6a**	2-OC_4_H_9_	5.60	86.67	53.42	763	763	763	763	373	745	>30	182
**6b**	3-OC_4_H_9_	5.54	86.67	53.42	763	763	763	763	745	745	16.5 ± 0.8	7.8
**6c**	4-OC_4_H_9_	5.60	86.67	53.42	763	763	763	763	745	745	>30	ND
**7a**	2-OCH(CH_3_)_2_	5.18	70.54	53.79	796	796	796	796	389	778	>30	138
**7b**	3-OCH(CH_3_)_2_	5.13	70.54	53.79	796	796	796	796	**24**	778	27.4 ± 0.6	6.9
**7c**	4-OCH(CH_3_)_2_	5.11	70.54	53.79	796	796	796	796	389	778	>30	ND
**8a**	2-OCH(CH_3_)C_2_H_5_	5.52	87.05	52.38	**5.9**	**11.9**	**11.9**	**11.9**	**23**	**89**	>30	134
**8b**	3-OCH(CH_3_)C_2_H_5_	5.47	87.05	52.38	763	763	763	763	**23**	745	2.7 ± 0.7	8.3
**8c**	4-OCH(CH_3_)C_2_H_5_	5.46	87.05	52.38	763	763	763	763	89	745	>30	ND
**APC**	–	−	–	–	5.7	>46	>46	>46	–	–	–	–
**RIF**	–	−	–	–	–	–	–	–	10	109	–	–
**DCMU**	–	−	−	−	–	–	–	–	–	–	–	1.9

*^a^* calculated using ACD/Percepta ver. 2012; SA *= S. aureus* ATCC 29213, MRSA *=* clinical isolates of methicillin-resistant *S. aureus* 63718, SA 630 and 3202; MT = *M. tuberculosis* H37Ra, MAP = clinical isolate of *M. avium* subsp. *paratuberculosis* CIT03; ND = not determined due to precipitation.

### 2.2. In Vitro Antibacterial Susceptibility Testing

The *in vitro* antibacterial activity of the discussed compounds was evaluated against three clinical isolates of MRSA and *S. aureus* ATCC 29213 as a reference and quality control strain. All the compounds showed only moderate or negligible activity, except methoxy derivative **3a** [11], propoxy derivative **5a** and *sec*-butoxy one **8a**. The activity of compound **3a** is comparable with that of the standard; the activity of derivatives **5a** and **8a** is almost 4-fold higher than that of ampicillin. Based on the results presented in Table 1 it seems that the antibacterial efficacy is closely connected with *ortho*-substitution of the aniline ring and with the even-numbered length alkoxy chain. It is positively influenced by higher lipophilicity as well as increasing surface activity (*i.e.*, decreasing surface tension) of the compounds. The lipophilicity of the discussed compounds is influenced by the length and branching of the alkoxy tail of the R substituent, and it seems that anti-*Staphylococcus* potency is affected also by increasing bulkiness/molar volume of the alkoxy chain.

### 2.3. In Vitro Antimycobacterial Evaluation

The evaluation of the *in vitro* antimycobacterial activity of the compounds was performed against *M. tuberculosis* H37Ra ATCC 25177 [25] and clinical isolate of *M. avium* subsp. *paratuberculosis* CIT03 [26], see Table 1. The activity of compounds was expressed as the minimum inhibitory concentration (MIC) that is defined for mycobacteria as a 90% or greater (IC_90_) reduction of growth in comparison with the control. The MIC/IC_90_ value is routinely and widely used in bacterial assays, being a standard detection limit according to the Clinical and Laboratory Standards Institute [27].

Most of compounds did not show any antimycobacterial activity, except *ortho*- and *meta*-position isomers **8a**,**b** (R = 2-, 3-OCH(CH_3_)C_2_H_5_) and compound **7b** (R = 3-OCH(CH_3_)_2_) that demonstrated the highest activity against *M. tuberculosis* (MIC = 23 µM for **8a**,**b** and 24 µM for **7b**) within the discussed series of compounds. Compound **8a** (R = 2-OCH(CH_3_)C_2_H_5_) also exhibited higher activity against *M. avium* subsp. *paratuberculosis* than rifampicin. Due to limited number of the effective compounds it is not possible to formulate a more comprehensive SAR hypothesis. Based on the results presented in Table 1 it seems that the activity is positively influenced by increasing surface activity of the compounds and substitution in the *ortho*- and *meta*-position, especially by branched chains. An increasing antimycobacterial activity caused by branched chains was described recently [28,29].

Additionally, a standard MTT assay was performed on the selected most effective compounds, the MICs of which were previously determined through Alamar Blue assays (see Table 1). The MTT assay is a well-characterized method of assessing cell growth through measurement of respiration. For the purpose of this assay, an MTT measured viability of *M. tuberculosis* H37Ra of less than 70% after exposure to the MIC of each tested compound was considered a positive result. As such, a low level of cell viability may suggest inhibition of cell growth through respiratory inhibition [30]. All the selected compounds, *i.e.*, 3-isopropoxy (**7b**, 10.7%), 2-*sec*-butoxy (**8a**, 23.9%) and 3-*sec*-butoxy (**8b**, 21.7%) derivatives showed less than 70% viability of *M. tuberculosis* H37Ra at the lowest tested concentration (8 µg/mL, *i.e.*, *ca*. 24 µM).

Janin [31] discusses a similar type of carboxamides in his review and suggested the hypothesis that all these compounds can interfere with the mycobacterial proton pump F_0_F_1_H^+^ATPase or inhibit biosynthesis of amino acids. Based on the fact that the change in the colour of Alamar Blue is caused by a decrease of mycobacterial cell metabolism, it may be hypothesized that the mechanism of action of these *N*-alkoxyphenylamides of 3-hydroxynaphthalene-2-carboxylic acid could be connected with an effect on mycobacterial energy metabolism [32], however, we cannot rule out the possibility that the studied compounds acted upon a salicylanilides-like site present in the mycobacteria, as mentioned above ([7,8] and refs. therein).

### 2.4. In Vitro Cytotoxicity Assay

The preliminary *in vitro* screening of the cytotoxicity of the compounds was performed using the human monocytic leukemia THP-1 cell line. The cytotoxicity was evaluated as the LD_50_ value (LD_50_—lethal dose to 50% of the cell population), see Table 1. A compound is considered cytotoxic when it demonstrates a toxic effect on cells at concentrations up to 10 μM [33], and the highest tested concentration that was used for the toxicity assay was three times this value. Treatment with 30 μM by a majority of compounds did not lead to significant lethal effects on THP-1 cells. Compound **7b** (R = 3-OCH(CH_3_)_2_) demonstrated low toxicity (LD_50_ = 27.5 ± 0.5 µM) against THP-1. Compound **6b** (R = 3-OC_4_H_9_) showed an LD_50_ of 16.5 ± 0.8 µM, while **8b** (R = 3-OCH(CH_3_)C_2_H_5_) exerted fairly high toxicity (LD_50_ = 2.7 ± 0.7 µM; e.g., the LD_50_ of oxaliplatin against this cell line was formerly measured as 1.7 ± 0.6 µM). It can be stated that cytotoxicity is closely connected with the substitution of the *meta*-position of aniline and increases with a longer and branched tail, *i.e.*, it seems that cytotoxicity increases with increasing bulkiness of the alkoxy chain as well as increasing surface activity of the compounds (*i.e.*, surface tension decrease), see **5b** (LD_50_ >30 μM, MV = 70.16 cm^3^, ST = 54.92 dyne/cm) < **7b** (LD_50_ = 27.5 µM, MV = 70.54 cm^3^, ST = 53.79 dyne/cm) << **6b** (LD_50_ = 16.5 µM, MV = 86.67 cm^3^, ST = 53.42 dyne/cm) <<< **8b** (LD_50_ = 2.7 µM, MV = 87.05 cm^3^, ST = 52.38 dyne/cm). Based on these observations it can be concluded that anilides with LD_50_ > 30 μM **3a**, **5a**, **4b**, **5b** and especially the most potent **8a** can be considered as promising agents for subsequent design of novel antibacterial and antimycobacterial agents, respectively.

### 2.5. Inhibition of Photosynthetic Electron Transport (PET) in Spinach Chloroplasts

As it was found that antimicrobial activity correlates with herbicidal effect, all the studied compounds were additionally evaluated also for their activity related to photosynthetic electron transport (PET) inhibition in spinach chloroplasts, which was reflected in the inhibition of photoreduction of artificial electron acceptor 2,6-dichlorophenol-indophenol. The PET inhibiting activity of the compounds was expressed by IC_50_ values. The most active compounds were *meta*-substituted derivatives. The aqueous solubility of the *para*-substituted compounds was limited; therefore IC_50_ values could not be determined due to precipitation of compounds during experiment. Compounds **4b** (R = 3-OC_2_H_5_; IC_50_ = 4.5 μM), **5b** (R = 3-OC_3_H_7_; IC_50_ = 4.8 μM) and **7b** (R = 3-OCH(CH_3_)_2_; IC_50_ = 6.9 μM) are effective PET inhibitors (IC_50_ of the standard DCMU was 1.9 μM).

**Figure 1:**
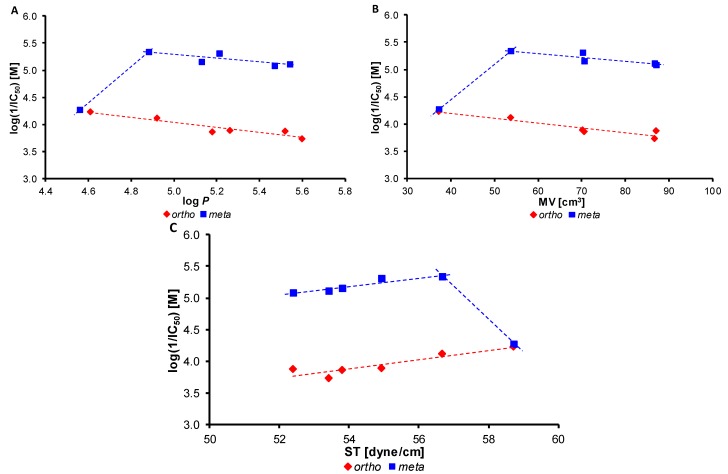
Dependence of PET inhibition log(1/IC_50_)[M] of tested compounds on lipophilicity expressed as log *P* (**A**); molar volume (MV [cm^3^]) of individual alkoxy chains (**B**); and surface tension (ST [dyne/cm]) (**C**).

The dependences of PET-inhibiting activity expressed as log(1/IC_50_) on the lipophilicity (log *P*) of the compounds, surface tension (ST [dyne/cm]) as well as on the molar volume (MV [cm^3^]) of individual alkoxy chains are illustrated in Figure 1A–C. The dependence of the PET-inhibiting activity of the *ortho*-substituted derivatives on the lipophilicity of the compounds and the molar volume of individual alkoxy tails linearly decreased (*r* = −0.9454, *n* = 6 and *r* = −0.9439, *n* = 6), see Figure 1A,B. The PET inhibition linearly increased with increasing surface tension, *i.e.*, decreasing surface activity, see Figure 1C.

On the other hand, the dependences of the PET-inhibiting activity of *meta*-substituted compounds on physicochemical parameters were biphasic. The PET inhibition rapidly increased from log *P* = 4.56 (R = 3-OCH_3_, **3b**) to log *P* = 4.88 (R = 3-OC_2_H_5_, **4b**) and with the further increase of compound lipophilicity and the bulkiness of the alkoxy chain it slowly decreased indicating that the prolongation and branching of the alkoxy chain is connected with an activity drop. Also the PET inhibition linearly increased with increasing surface tension (*i.e.*, with surface activity decreasing) to ST = 56.66 dyne/cm (compound **4b**) and then rapidly decreased up to ST = 58.71 dyne/cm (compound **3b**), see Figure 1C.

It is important to note that, while the increase in the antimicrobial activity and the cytotoxicity is connected especially with the extension of the alkoxy tail and the increase in surface activity, effective PET inhibitors are typically characterized by lower surface activity and rather short and unbranched alkoxy chains. However, it should be mentioned that although both methoxy derivatives **3a** and **3b** showed similar PET inhibition and were characterized by similar calculated parameters, compound **3a** was the most effective within the *ortho*-substituted series, while compound **3b** was the least potent within the *meta*-substituted series.

By the addition of 1,5-diphenylcarbazide (DPC), an artificial electron donor acting in Z^●^/D^●^ intermediate on the donor side of PS II [34], to chloroplasts treated with the studied compounds, in which PET was inhibited by about 85%–94%, PET was practically completely restored only in the presence of very high DPC concentrations (approx. 2.3 mM). As DPC can alter the binding of compounds with herbicidal activity, e.g., atrazin or metribuzin, presumably due to overlapping binding domain in the Q_B_ pocket but its effect on the Q_B_ site can affect plastoquinone reduction only at relatively high concentrations (>2 M) [35,36], it could be assumed that the inhibitory site of action of the studied compounds is situated on the acceptor side of PS II, in the section at Q_B_ site. Previously it was found that PET on the acceptor side of PS II was partially damaged also by ring-substituted salicylanilides and carbamoylphenylcarbamates [37], ring-substituted 4-arylamino-7-chloroquinolinium chlorides [38], 5-*tert*-butyl-6-chloro-*N*-(3-fluorophenyl)pyrazine-2-carboxamide and 5-*tert*-butyl-*N*-(3-hydroxy-4-chlorophenyl)pyrazine-2-carboxamide [39] and by *N*-benzylpyrazine-2-carboxamides [40].

**Figure 2:**
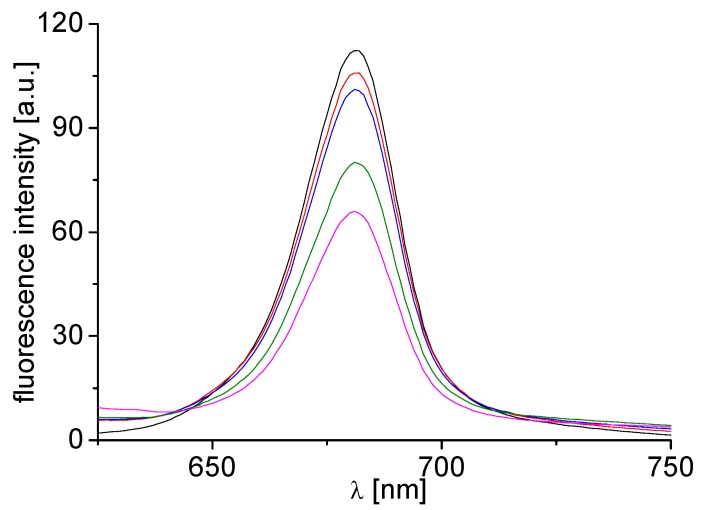
Fluorescence emission spectra of chlorophyll *a* in untreated spinach chloroplasts in presence of compound **4b**: 0, 102, 204, 408 and 612 μM (curves from top to bottom); λex = 436 nm.

The studied compounds affected chlorophyll *a* (Chl*a*) fluorescence in spinach chloroplasts indicating their interactions with constituents of photosynthetic apparatus. Fluorescence emission spectra of Chl*a* in spinach chloroplasts treated with compound **4b** are shown in Figure 2. The decreased intensity of the emission band at 686 nm belonging to the Chl*a-*protein complexes occurring mainly in photosystem II [41] suggested PS II as the site of action of the studied inhibitors. The extent of perturbation of Chl*a-*protein complexes in the thylakoid membrane is reflected in the sharpness of decreased fluorescence of Chl*a* pigment. A similar decrease of Chl*a* fluorescence in plant chloroplasts was also observed previously after treatment with ring-substituted 1-hydroxynaphthalene-2-carboxanilides [17], 2-hydroxynaphthalene-1-carboxanilides [18], 5-bromo- and 3,5-dibromo-2-hydroxy-*N*-phenyl-benzamides [42] and ring-substituted 4-arylamino-7-chloroquinolinium chlorides [38].

## 3. Experimental Section

### 3.1. General Information

All reagents were purchased from Sigma-Aldrich (St. Louis, MO, USA) and Merck (Darmstadt, Germany). Reactions were carried out in StartSYNTH microwave labstation (Milestone, Sorisole, Italy). TLC experiments were performed on alumina‑backed silica gel 40 F254 plates (Merck). The plates were illuminated under UV (254 nm) and evaluated in iodine vapour. The melting points were determined on Kofler hot-plate apparatus HMK (Franz Kustner Nacht KG, Dresden, Germany) and are uncorrected. Infrared (IR) spectra were recorded on a Smart MIRacle™ ATR ZnSe for Nicolet™ Impact 410 FT-IR spectrometer (Thermo Electron Corporation, West Palm Beach, FL, USA). All ^1^H- and ^13^C-NMR spectra were recorded on an Avance III 400 MHz FT-NMR spectrometer (Bruker, Karlsruhe, Germany) in DMSO-*d*_6_. ^1^H and ^13^C chemical shifts (δ) are reported in ppm using signal of the solvent as a standard (2.500 and 39.50 ppm). Mass spectra were measured using a LTQ Orbitrap Hybrid Mass Spectrometer (Thermo Electron Corporation) with direct injection into an APCI source (400 °C) in the positive mode. Lipophilicity (log *P*) of the final compounds, surface tension and molar volume of R substituents were predicted using ACD/Percepta ver. 2012 (Advanced Chemistry Development, Inc., Toronto, ON, Canada).

### 3.2. Synthesis

#### 3.2.1. General Procedure for the Synthesis of Anilines **1a**–**1o**

These syntheses were carried out in one step as per De Marco *et al.* [24] Aminophenol (92 mmol) and a suspension of NaH in mineral oil (275 mmol NaH) were suspended in dry DMF (100 mL). The corresponding alkylbromide (137 mmol) was added dropwise and the mixture was then stirred for 24 h at room temperature. The reaction was quenched by adding 400 mL of distilled water and the aqueous layer was extracted with 3 × 150 mL of ethyl acetate. Collected organic layers were dried over anhydrous Na_2_SO_4_ and concentrated *in vacuo*. Pure product was obtained by distillation under reduced pressure.

*2-Ethoxyaniline* (**1a**). Yield 70%; bp. 109–111 °C (10 mmHg); ^1^H-NMR (DMSO-*d*_6_), δ: 6.76 (d, *J* = 8.0 Hz, 1H); 6.61–6.66 (m, 2H); 6.47–6.54 (m, 1H); 4.62 (s, 2H); 3.97 (q, *J* = 7.0 Hz, 2H); 1.33 (t, *J* = 7.0 Hz, 3H); ^13^C-NMR (DMSO-*d*_6_), δ: 145.53, 137.78, 120.88, 116.25, 113.96, 111.79, 63.28, 14.86.

*3-Ethoxyaniline* (**1b**). Yield 80%; bp. 125–127 °C (10 mmHg); ^1^H-NMR (DMSO-*d*_6_), δ: 6.88 (t, *J* = 8.4 Hz, 1H); 6.11–6.16 (m, 2H); 6.02–6.08 (m, 1H); 5.00 (s, 2H); 3.89 (q, *J* = 7.0 Hz, 2H); 1.28 (t, *J* = 7.0 Hz, 3H); ^13^C-NMR (DMSO-*d*_6_), δ: 159.55, 149.96, 129.54, 106.78, 102.08, 99.97, 62.38, 14.81.

*4-Ethoxyaniline* (**1c**). Yield 78%; bp: 126–128 °C (10 mmHg) [43]; ^1^H-NMR (DMSO-*d*_6_), δ: 6.63 (d, *J* = 8.8 Hz, 2H); 6.49 (d, *J* = 8.8 Hz, 2H); 4.57 (s, 2H); 3.85 (q, *J* = 7.0 Hz, 2H); 1.25 (t, *J* = 7.0 Hz, 3H); ^13^C-NMR (DMSO-*d*_6_), δ: 149.86, 142.29, 115.29, 114.99, 63.31, 14.90.

*2-Propoxyaniline* (**1d**). Yield 63%; bp. 127–129 °C (10 mmHg); ^1^H-NMR (DMSO-*d*_6_), δ: 6.75 (d, *J* = 8.0 Hz, 1H); 6.62–6.67 (m, 2H); 6.49–6.54 (m, 1H); 4.62 (s, 2H); 3.87 (t, *J* = 6.6 Hz, 2H); 1.73 (sx, *J* = 7.0 Hz, 2H); 0.99 (t, *J* = 7.3 Hz, 3H); ^13^C-NMR (DMSO-*d*_6_), δ: 145.165, 137.75, 120.82, 116.28, 113.96, 111.60, 69.16, 22.25, 10.56.

*3-Propoxyaniline* (**1e**). Yield 73%; bp. 125–127 °C (10 mmHg); ^1^H-NMR (DMSO-*d*_6_), δ: 6.87 (t, *J* = 8.4 Hz, 1H); 6.11–6.15 (m, 2H); 6.03–6.08 (m, 1H); 5.00 (s, 2H); 3.79 (t, *J* = 6.6 Hz, 2H); 1.67 (sx, *J* = 7.0 Hz, 2H); 0.95 (t, *J* = 7.3 Hz, 3H); ^13^C-NMR (DMSO-*d*_6_), δ: 159.69, 149.95, 129.51, 106.73, 102.08, 100.01, 68.39, 22.16, 10.46.

*4-Propoxyaniline* (**1f**). Yield 73%; bp. 129–131 °C (10 mmHg); ^1^H-NMR (DMSO-*d*_6_), δ: 6.63 (d, *J* = 8.8 Hz, 2H); 6.49 (d, *J* = 8.8 Hz, 2H); 4.57 (s, 2H); 3.76 (t, *J* = 6.6 Hz, 2H); 1.65 (sx, *J* = 7.0 Hz, 2H); 0.94 (t, *J* = 7.3 Hz, 3H); ^13^C-NMR (DMSO-*d*_6_), δ: 149.98, 142.19, 115.32, 114.90, 69.45, 22.16, 10.35.

*2-Butoxyaniline* (**1g**). Yield 68%; bp. 133–135 °C (10 mmHg); ^1^H-NMR (DMSO-*d*_6_), δ: 6.75 (d, *J* = 8.0 Hz, 1H); 6.62–6.68 (m, 2H); 6.48–6.53 (m, 1H); 4.62 (s, 2H); 3.90 (t, *J* = 6.4 Hz, 2H); 1.70 (qi, *J* = 7.2 Hz, 2H); 1.43 (sx, *J* = 7.3 Hz, 2H); 0.94 (t, *J* = 7.3 Hz, 3H); ^13^C-NMR (DMSO-*d*_6_), δ: 145.71, 137.71, 120.80, 116.25, 113.96, 111.60, 67.35, 31.19, 19.10, 13.73.

*3-Butoxyaniline* (**1h**). Yield 70%; bp. 136–139 °C (10 mmHg); ^1^H-NMR (DMSO-*d*_6_), δ: 6.87 (t, *J* = 8.4 Hz, 1H); 6.11–6.14 (m, 2H); 6.03–6.08 (m, 1H); 5.00 (s, 2H); 3.83 (t, *J* = 6.4 Hz, 2H); 1.65 (qi, *J* = 7.2 Hz, 2H); 1.40 (sx, *J* = 7.3 Hz, 2H); 0.91 (t, *J* = 7.3 Hz, 3H); ^13^C-NMR (DMSO-*d*_6_), δ: 159.71, 149.93, 129.49, 106.73, 102.08, 100.01, 66.55, 30.90, 18.80, 13.72.

*4-Butoxyaniline* (**1i**). Yield 79%; bp. 132–135 °C (10 mmHg); ^1^H-NMR (DMSO-*d*_6_), δ: 6.63 (d, *J* = 8.8 Hz, 2H); 6.49 (d, *J* = 8.8 Hz, 2H); 4.57 (s, 2H); 3.79 (t, *J* = 6.4 Hz, 2H); 1.62 (qi, *J* = 7.2 Hz, 2H); 1.39 (sx, *J* = 7.3 Hz, 2H); 0.91 (t, *J* = 7.3 Hz, 3H); ^13^C-NMR (DMSO-*d*_6_), δ: 150.04, 142.27, 115.34, 114.97, 67.59, 31.03, 18.80, 13.75.

*2-(Prop-2-yloxy)aniline* (**1j**). Yield 52%; bp. 107–110 °C (10 mmHg); ^1^H-NMR (DMSO-*d*_6_), δ: 6.78 (d, *J* = 8.0 Hz, 1H); 6.63–6.66 (m, 2H); 6.48–6.54 (m, 1H); 4.60 (s, 2H); 4.45 (septet, *J* = 6.0 Hz, 1H); 1.25 (d, *J* = 6.0 Hz, 6H); ^13^C-NMR (DMSO-*d*_6_), δ: 144.23, 138.76, 120.90, 116.26, 114.29, 113.80, 70.23, 22.03.

*3-(Prop-2-yloxy)aniline* (**1k**). Yield 56%; bp. 118–120 °C (10 mmHg); ^1^H-NMR (DMSO-*d*_6_), δ: 6.86 (t, *J* = 8.4 Hz, 1H); 6.10–6.13 (m, 2H); 6.02–6.07 (m, 1H); 4.98 (s, 2H); 4.43 (septet, *J* = 6.0 Hz, 1H); 1.21 (d, *J* = 6.0 Hz, 6H); ^13^C-NMR (DMSO-*d*_6_), δ: 158.45, 149.99, 129.56, 106.70, 103.29, 101.30, 68.53, 22.04.

*4-(Prop-2-yloxy)aniline* (**1l**). Yield 62%; bp. 130–132 °C (10 mmHg); ^1^H-NMR (DMSO-*d*_6_), δ: 6.62 (d, *J* = 8.8 Hz, 2H); 6.48 (d, *J* = 8.8 Hz, 2H); 4.59 (s, 2H); 4.30 (septet, *J* = 6.0 Hz, 1H); 1.17 (d, *J* = 6.0 Hz, 6H); ^13^C-NMR (DMSO-*d*_6_), δ: 148.51, 142.54, 117.46, 114.99, 70.08, 22.07.

*2-(But-2-yloxy)aniline* (**1m**). Yield 56%; bp. 112–113 °C (10 mmHg); ^1^H-NMR (DMSO-*d*_6_), δ: 6.76 (d, *J* = 8.0 Hz, 1H); 6.62–6.66 (m, 2H); 6.46–6.52 (m, 1H); 4.56 (s, 2H); 4.24 (sx, *J* = 6.0 Hz, 1H); 1.48–1.70 (m, 2H); 1.20 (d, *J* = 6.2 Hz, 3H); 0.92 (t, *J* = 7.3 Hz, 3H); ^13^C-NMR (DMSO-*d*_6_), δ: 144.44, 138.77, 120.92, 116.26, 114.29, 113.82, 74.85, 28.68, 19.23, 9.59.

*3-(But-2-yloxy)aniline* (**1n**). Yield 60%; bp. 120–123 °C (10 mmHg); ^1^H-NMR (DMSO-*d*_6_), δ: 6.86 (t, *J* = 8.4 Hz, 1H); 6.09–6.13 (m, 2H); 6.02–6.08 (m, 1H); 4.98 (s, 2H); 4.20 (sx, *J* = 6.0 Hz, 1H); 1.45–1.65 (m, 2H); 1.18 (d, *J* = 6.2 Hz, 3H); 0.89 (t, *J* = 7.3 Hz, 3H); ^13^C-NMR (DMSO-*d*_6_), δ: 158.79, 149.99, 129.56, 106.70, 103.32, 101.35, 73.61, 28.73, 19.27, 9.65.

*4-(But-2-yloxy)aniline* (**1o**). Yield 64%; bp. 132–133 °C (10 mmHg); ^1^H-NMR (DMSO-*d*_6_), δ: 6.62 (d, *J* = 8.8 Hz, 2H); 6.48 (d, *J* = 8.8 Hz, 2H); 4.59 (s, 2H); 4.06 (sx, *J* = 6.0 Hz, 1H); 1.45–1.65 (m, 2H); 1.13 (d, *J* = 6.2 Hz, 3H); 0.89 (t, *J* = 7.3 Hz, 3H); ^13^C-NMR (DMSO-*d*_6_), δ: 148.83, 142.51, 117.49, 114.97, 75.26, 28.67, 19.24, 9.62.

#### 3.2.2. General Procedure for the Synthesis of Carboxamide Derivatives **2**–**8c**

3-Hydroxynaphthalene-2-carboxylic acid (5.3 mmol) and the appropriate alkoxyaniline (5.3 mmol) were suspended in dry chlorobenzene (25 mL). Phosphorous trichloride (0.23 mL, 2.7 mmol) was added dropwise, and the reacting mixture was heated in the microwave reactor at maximal allowed power 500 W and 130 °C, using infrared flask-surface control of temperature, for 15 min. The solvent was evaporated under reduced pressure and the solid residue washed with 50 mL of 2 M HCl. The crude product was recrystallized from aqueous ethanol.

*3-Hydroxy-N-phenylnaphthalene-2-carboxamide* (**2**), *3-hydroxy-N-(2-methoxyphenyl)naphthalene-2-carboxamide* (**3a**), *3-hydroxy-N-(3-methoxyphenyl)naphthalene-2-carboxamide* (**3b**) and *3-hydroxy-N-(4-methoxyphenyl)naphthalene-2-carboxamide* (**3c**) were synthesized and characterized recently [11].

*N-(2-Ethoxyphenyl)-3-hydroxynaphthalene-2-carboxamide* (**4a**). Yield 61%; Mp. 157–160 °C; IR (Zn/Se ATR, cm^−^^1^): 3418, 2974, 2888, 1662, 1603, 1589, 1538, 1455, 1441, 1393, 1343, 1312, 1293, 1249, 1216, 1142, 1116, 1033, 924, 871, 789, 742; ^1^H-NMR (DMSO-*d*_6_), δ: 11.67 (s, 1H), 11.19 (s, 1H), 8.72 (s, 1H), 8.56 (d, *J* = 7.0 Hz, 1H), 7.99 (d, *J* = 8.1 Hz, 1H), 7.79 (d, *J* = 8.1 Hz, 1H), 7.52 (td, *J* = 7.0 Hz, 1.5 Hz, 1H), 7.30–7.40 (m, 2H), 7.06–7.09 (m, 2H), 6.93–7.04 (m, 1H), 4.15 (q, *J* = 7.0 Hz, 2H), 1.47 (t, *J* = 7.0 Hz, 3H); ^13^C-NMR (DMSO-*d*_6_), δ: 162.54, 152.54, 147.67, 135.77, 132.63, 128.93, 128.32, 128.17, 127.19, 125.59, 123.77, 123.68, 121.36, 120.56, 119.72, 111.85, 110.69, 64.14, 14.71; HR-MS: for C_19_H_17_NO_3_ [M+H]^+^ calculated 308.12812 *m*/*z*, found 308.12860 *m*/*z*.

*N-(3-Ethoxyphenyl)-3-hydroxynaphthalene-2-carboxamide* (**4b**).Yield 56%; Mp. 173–175 °C; IR (Zn/Se ATR, cm^−^^1^): 2973, 1623, 1593, 1549, 1532, 1458, 1399, 1362, 1266, 1215, 1178, 1158, 1133, 1109, 1050, 921, 882; ^1^H-NMR (DMSO-*d*_6_), δ: 11.30 (s, 1H), 10.55 (s, 1H), 8.48 (s, 1H), 7.93 (d, *J* = 7.7 Hz, 1H), 7.77 (d, *J* = 8.4 Hz, 1H), 7.47–7.54 (m, 2H), 7.26–7.40 (m, 4H), 6.68–6.74 (m, 1H), 4.03 (q, *J* = 7.0 Hz, 2H), 1.35 (t, *J* = 7.0 Hz, 3H); ^13^C-NMR (DMSO-*d*_6_), δ: 165.59, 158.76, 153.65, 139.55, 135.71, 130.40, 129.48, 128.63, 128.05, 126.81, 125.70, 123.68, 121.77, 112.57, 110.51, 110.02, 106.73, 62.96, 14.57; HR-MS: for C_19_H_17_NO_3_ [M+H]^+^ calculated 308.12812 *m*/*z*, found 308.12869 *m*/*z*.

*N-(4-Ethoxyphenyl)-3-hydroxynaphthalene-2-carboxamide* (**4c**). Yield 49%; Mp. 221–224 °C; IR (Zn/Se ATR, cm^−^^1^): 3052, 2975, 2929, 1635, 1619, 1609, 1564, 1558, 1510, 1394, 1357, 1346, 1247, 1214, 1172, 1146, 1119,1070, 1047, 949; ^1^H-NMR (DMSO-*d*_6_), δ: 11.47 (s, 1H), 10.51 (s, 1H), 8.53 (s, 1H), 7.92 (d, *J* = 8.1 Hz, 1H), 7.77 (d, *J* = 8.4 Hz, 1H), 7.66 (d, *J* = 8.8 Hz, 2H), 7.51 (td, *J* = 7.0 Hz, 1.5 Hz, 1H), 7.36–7.40 (m, 1H), 7.32 (s, 1H), 6.95 (d, *J* = 9.2 Hz, 2H), 4.02 (q, *J* = 7.0 Hz, 2H), 1.33 (t, *J* = 7.0 Hz, 3H); ^13^C-NMR (DMSO-*d*_6_), δ: 165.59, 155.15, 154.15, 135.76, 131.15, 130.08, 128.61, 128.04, 126.75, 125.70, 123.64, 122.27, 120.93, 114.39, 110.56, 63.09, 14.59; HR-MS: for C_19_H_17_NO_3_ [M+H]^+^ calculated 308.12812 *m*/*z*, found 308.12857 *m*/*z*.

*3-Hydroxy-N-(2-propoxyphenyl)naphthalene-2-carboxamide* (**5a**). Yield 89%; Mp. 164–166 °C; IR (Zn/Se ATR, cm^−^^1^): 3432, 2964, 2936, 2874, 1652, 1607, 1538, 1520, 1456, 1446, 1343, 1310, 1293, 1215, 1172, 1149, 1115, 1041, 1018, 873; ^1^H-NMR (DMSO-*d*_6_), δ: 11.62 (s, 1H), 11.11 (s, 1H), 8.72 (s, 1H), 8.55 (d, *J* = 7.4 Hz, 1H), 7.99 (d, *J* = 8.2 Hz, 1H), 7.77 (d, *J* = 8.0 Hz, 1H), 7.53 (td, *J* = 7.0 Hz, 1.2 Hz, 1H), 7. 41 (s, 2H), 7.32 (td, *J* = 7.0 Hz, 1.0 Hz, 2H), 6.93–7.09 (m, 1H), 4.05 (t, *J* = 6.6 Hz, 2H), 1.86 (sx, *J* = 7.0 Hz, 2H), 1.06 (t, *J* = 7.4 Hz, 3H); ^13^C-NMR (DMSO-*d*_6_), δ: 162.60, 152.59, 147.81, 135.78, 132.65, 128.93, 128.16, 128.16, 127.17, 125.59, 123.77, 123.77, 121.34, 120.43, 119.89, 111.66, 110.71, 69.89, 22.11, 10.44; HR-MS: for C_20_H_19_NO_3_ [M+H]^+^ calculated 322.14377 *m*/*z*, found 322.14435 *m*/*z*.

*3-Hydroxy-N-(3-propoxyphenyl)naphthalene-2-carboxamide* (**5b**). Yield 71%; Mp. 170–173 °C; IR (Zn/Se ATR, cm^−^^1^): 2962, 2936, 2899, 2870, 1635, 1620, 1591, 1549, 1538, 1447, 1394, 1343, 1257, 1223, 1211, 1157, 1048, 1027; ^1^H-NMR (DMSO-*d*_6_), δ: 11.29 (s, 1H), 10.55 (s, 1H), 8.48 (s, 1H), 7.94 (d, *J* = 8.7 Hz, 1H), 7.77 (d, *J* = 8.7 Hz, 1H), 7.51 (td, *J* = 6.6 Hz, 1.5 Hz, 1H), 7.48 (s, 1H), 7.26–7.40 (m, 4H), 6.70–6.74 (m, 1H), 3.93 (t, *J* = 6.6 Hz, 2H), 1.75 (sx, *J* = 7.0 Hz, 2H), 0.99 (t, *J* = 7.3 Hz, 3H); ^13^C-NMR (DMSO-*d*_6_), δ: 165.60, 158.93, 153.66, 139.55, 135.71, 130.40, 129.48, 128.63, 128.05, 126.81, 125.71, 123.68, 121.75, 112.57, 110.53, 110.07, 106.76, 68.91, 21.96, 10.31; HR-MS: for C_20_H_19_NO_3_ [M+H]^+^ calculated 322.14377 *m*/*z*, found 322.14420 *m*/*z*.

*3-Hydroxy-N-(4-propoxyphenyl)naphthalene-2-carboxamide* (**5c**). Yield 66%; Mp. 219–221 °C; IR (Zn/Se ATR, cm^−^^1^): 2975, 2962, 2926, 1653, 1616, 1559, 1506, 1473, 1451, 1391, 1358, 1348, 1239, 1210, 1170, 1068, 981, 951, 913; ^1^H-NMR (DMSO-*d*_6_), δ: 11.48 (s, 1H), 10.51 (s, 1H), 8.54 (s, 1H), 7.92 (d, *J* = 8.4 Hz, 1H), 7.77 (d, *J* = 8.4 Hz, 1H), 7.66 (d, *J* = 9.2 Hz, 2H), 7.51 (td, *J* = 6.6 Hz, 1.1 Hz, 1H), 7.36 (td, *J* = 5.1 Hz, 1.10 Hz, 1H), 7.33 (s, 1H), 6.95 (d, *J* = 9.2 Hz, 2H), 3.92 (t, *J* = 6.6 Hz, 2H), 1.73 (sx, *J* = 6.6 Hz, 2H), 0.98 (t, *J* = 7.3 Hz, 3H); ^13^C-NMR (DMSO-*d*_6_), δ: 165.60, 155.32, 154.16, 135.76, 131.15, 130.11, 128.61, 128.05, 126.75, 125.70, 123.64, 122.27, 120.90, 114.44, 110.57, 69.07, 21.98, 10.29; HR-MS: for C_20_H_19_NO_3_ [M+H]^+^ calculated 322.14377 *m*/*z*, found 322.14429 *m*/*z*.

*N-(2-Butoxyphenyl)-3-hydroxynaphthalene-2-carboxamide* (**6a**). Yield 63%; Mp. 156–159 °C; IR (Zn/Se ATR, cm^−^^1^): 3143, 2956, 2926, 2872, 1637, 1622, 1609, 1594, 1559, 1549, 1492, 1456, 1394, 1358, 1343, 1287, 1251, 1222, 1208, 1116, 1065, 1035, 1006, 864; ^1^H-NMR (DMSO-*d*_6_), δ: 11.59 (s, 1H), 11.09 (s, 1H), 8.72 (s, 1H), 8.54 (d, *J* = 7.4 Hz, 1H), 7.99 (d, *J* = 8.1 Hz, 1H), 7.79 (d, *J* = 8.1 Hz, 1H), 7.53 (td, *J* = 7.0 Hz, 1.5 Hz, 1H), 7.41 (s, 1H), 7.36 (td, *J* = 7.0 Hz, 1.5 Hz, 1H), 7.09–6.97 (m, 3H), 4.08 (t, *J* = 6.6 Hz, 2H), 1.83 (qi, *J* = 6.6 Hz, 2H), 1.50 (sx, *J* = 7.3 Hz, 2H), 0.97 (t, *J* = 7.3 Hz, 3H); ^13^C-NMR (DMSO-*d*_6_), δ: 162.63, 152.62, 147.84, 135.82, 132.71, 128.99, 128.25, 128.17, 127.20, 125.65, 123.84, 123.82, 121.36, 120.49, 119.92, 111.71, 110.77, 68.04, 30.97, 18.97, 13.65; HR-MS: for C_21_H_21_NO_3_ [M+H]^+^ calculated 336.15942 *m*/*z*, found 336.15980 *m*/*z*.

*N-(3-Butoxyphenyl)-3-hydroxynaphthalene-2-carboxamide* (**6b**). Yield 56%; Mp. 153–155 °C; IR (Zn/Se ATR, cm^−^^1^): 2958, 2937, 2870, 1635, 1616, 1594, 1558, 1454, 1447, 1397, 1278, 1224, 1213, 1174, 1158, 1064, 1042, 1014, 977, 875, 857; ^1^H-NMR (DMSO-*d*_6_), δ: 11.30 (s, 1H), 10.55 (s, 1H), 8.48 (s, 1H), 7.93 (d, *J* = 8.1 Hz, 1H), 7.77 (d, *J* = 8.1 Hz, 1H), 7.51 (td, *J* = 6.1 Hz, 1.4 Hz, 1H), 7.47 (s, 1H), 7.22–7.39 (m, 4H), 6.68–6.74 (m, 1H), 3.97 (t, *J* = 6.2 Hz, 2H), 1.72 (qi, *J* = 6.6 Hz, 2H), 1.45 (sx, *J* = 7.7 Hz, 2H), 0.94 (t, *J* = 7.3 Hz, 3H); ^13^C-NMR (DMSO-*d*_6_), δ: 165.60, 158.94, 153.66, 139.54, 135.71, 130.37, 129.46, 128.63, 128.07, 126.81, 125.71, 123.68, 121.75, 112.57, 110.53, 110.07, 106.76, 67.10, 30.67, 18.67, 13.60; HR-MS: for C_21_H_21_NO_3_ [M+H]^+^ calculated 336.15942 *m*/*z*, found 336.15972 *m*/*z*.

*N-(4-Butoxyphenyl)-3-hydroxynaphthalene-2-carboxamide* (**6c**). Yield 63%; Mp. 211–214 °C; IR (Zn/Se ATR, cm^−^^1^): 3053, 2956, 2937, 2871, 1635, 1616, 1557, 1511, 1394, 1357, 1248, 1216, 1171, 1070, 1039, 950; ^1^H-NMR (DMSO-*d*_6_), δ: 11.47 (s, 1H), 10.50 (s, 1H), 8.53 (s, 1H), 7.92 (d, *J* = 8.1 Hz, 1H), 7.76 (d, *J* = 8.1 Hz, 1H), 7.65 (d, *J* = 9.2 Hz, 2H), 7.51 (td, *J* = 7.0 Hz, 1.5 Hz, 1H), 7.36 (td, *J* = 7.0 Hz, 1.5 Hz, 1H), 7.32 (s, 1H), 6.95 (d, *J* = 8.8 Hz, 2H), 3.96 (t, *J* = 6.6 Hz, 2H), 1.70 (qi, *J* = 8.1 Hz, 2H), 1.44 (sx, *J* = 8.1 Hz, 2H), 0.94 (t, *J* = 7.3 Hz, 3H); ^13^C-NMR (DMSO-*d*_6_), δ: 165.60, 155.35, 154.16, 135.79, 131.19, 130.14, 128.67, 128.11, 126.79, 125.76, 123.71, 122.27, 121.04, 114.46, 110.59, 67.28, 30.76, 18.71, 13.68; HR-MS: for C_21_H_21_NO_3_ [M+H]^+^ calculated 336.15942 *m*/*z*, found 336.15982 *m*/*z*.

*3-Hydroxy-N-[2-(prop-2-yloxy)phenyl]naphthalene-2-carboxamide* (**7a**). Yield 71%; Mp. 196–198 °C; IR (Zn/Se ATR, cm^−^^1^): 3335, 2981, 2972, 2913, 1652, 1635, 1605, 1592, 1539, 1486, 1453, 1409, 1373, 1340, 1284, 1221, 1176, 1118, 1069, 950, 924, 863, 835, 733, 690; ^1^H-NMR (DMSO-*d*_6_), δ: 11.65 (s, 1H), 11.19 (s, 1H), 8.72 (s, 1H), 8.58 (dd, *J* = 7.7 Hz, 1.8 Hz, 1H), 7.99 (d, *J* = 8.1 Hz, 1H), 7.79 (d, *J* = 8.4 Hz, 1H), 7.52 (td, *J* = 7.0 Hz, 1.5 Hz, 1H), 7.41 (s, 1H), 7.36 (td, *J* = 8.0 Hz, 1.8 Hz, 1H), 6.96–7.14 (m, 3H), 4.69–4.75 (m, 1H), 1.36 (d, *J* = 5.9 Hz, 6H); ^13^C-NMR (DMSO-*d*_6_), δ: 162.40, 152.51, 146.38, 135.77, 132.71, 129.45, 128.95, 128.16, 127.19, 125.59, 123.77, 123.57, 121.42, 120.65, 119.83, 113.64, 110.68, 71.01, 21.84; HR-MS: for C_20_H_19_NO_3_ [M+H]^+^ calculated 322.14377 *m*/*z*, found 322.14433 *m*/*z*.

*3-Hydroxy-N-[3-(prop-2-yloxy)phenyl]naphthalene-2-carboxamide* (**7b**). Yield 98%; Mp. 162–164 °C; IR (Zn/Se ATR, cm^−^^1^): 3024, 2970, 2909, 1637, 1622, 1586, 1557, 1532, 1452, 1257, 1221, 1170, 1152, 1116, 1001, 874, 776, 758, 740, 668; ^1^H-NMR (DMSO-*d*_6_), δ: 11.30 (s, 1H), 10.54 (s, 1H), 8.47 (s, 1H), 7.93 (d, *J* = 8.1 Hz, 1H), 7.77 (d, *J* = 8.4 Hz, 1H), 7.51 (td, *J* = 7.0 Hz, 1.5 Hz, 1H), 7.45 (s, 1H), 7.36 (td, *J* = 7.0 Hz, 1.5 Hz, 1H), 7.33 (s, 1H), 7.24–7.28 (m, 2H), 6.68–6.73 (m, 1H), 4.52–4.66 (m, 1H), 1.29 (d, *J* = 5.9 Hz, 6H); ^13^C-NMR (DMSO-*d*_6_), δ: 165.65, 157.76, 152.66, 139.69, 135.76, 130.45, 129.60, 128.72, 128.14, 126.88, 125.79, 123.77, 122.01, 112.51, 111.24, 110.54, 107.89, 69.25, 21.85; HR-MS: for C_20_H_19_NO_3_ [M+H]^+^ calculated 322.14377 *m*/*z*, found 322.14433 *m*/*z*.

*3-Hydroxy-N-[4-(prop-2-yloxy)phenyl]naphthalene-2-carboxamide* (**7c**). Yield 70%; Mp. 205–207 °C; IR (Zn/Se ATR, cm^−^^1^): 2978, 1653, 1602, 1558, 1505, 1452, 1346, 1237, 1210, 1172, 1120, 1108, 1070, 951, 871, 839, 826, 742, 712; ^1^H-NMR (DMSO-*d*_6_), δ: 11.47 (s, 1H), 10.50 (s, 1H), 8.52 (s, 1H), 7.92 (d, *J* = 7.7 Hz, 1H), 7.76 (d, *J* = 8.1 Hz, 1H), 7.64 (d, *J* = 8.8 Hz, 2H), 7.51 (t, *J* = 7.0 Hz, 1H), 7.36 (td, *J* = 7.3 Hz, 1.1 Hz, 1H), 7.32 (s, 1H), 6.94 (d, *J* = 8.8 Hz, 2H), 4.56–4.62 (m, 1H), 1.27 (d, *J* = 5.9 Hz, 6H); ^13^C-NMR (DMSO-*d*_6_), δ: 165.57, 154.13, 154.04, 135.74, 131.10, 130.10, 128.61, 128.04, 126.73, 125.70, 123.64, 122.28, 120.96, 115.78, 110.54, 69.38, 21.78; HR-MS: for C_20_H_19_NO_3_ [M+H]^+^ calculated 322.14377 *m*/*z*, found 322.14435 *m*/*z*.

*N-[2-(But-2-yloxy)phenyl]-3-hydroxynaphthalene-2-carboxamide* (**8a**). Yield 74%; Mp. 147–149 °C; IR (Zn/Se ATR, cm^−^^1^): 2971, 1653, 1634, 1616, 1592, 1538, 1488, 1454, 1398, 1340, 1287, 1252, 1221, 1127, 1117, 1067, 983, 921, 864, 836, 739; ^1^H-NMR (DMSO-*d*_6_), δ: 11.61 (s, 1H), 11.17 (s, 1H), 8.72 (s, 1H), 8.57 (dd, *J* = 7.7 Hz, 1.1 Hz, 1H), 7.99 (d, *J* = 8.1 Hz, 1H), 7.79 (d, *J* = 8.1 Hz, 1H), 7.52 (td, *J* = 7.0 Hz, 1.1 Hz, 1H), 7.41 (s, 1H), 7.36 (td, *J* = 7.0 Hz, 1.1 Hz, 1H), 6.91–7.12 (m, 3H), 4.51 (sx, *J* = 5.9 Hz, 1H), 1.57–1.90 (m, 2H), 1.31 (d, *J* = 5.9 Hz, 3H), 0.97 (t, *J* = 7.3 Hz, 3H); ^13^C-NMR (DMSO-*d*_6_), δ: 162.43, 152.52, 146.65, 135.76, 132.71, 129.25, 128.93, 128.16, 127.19, 125.59, 123.77, 123.60, 121.42, 120.46, 119.90, 113.27, 110.66, 75.78, 28.50, 19.12, 9.56; HR-MS: for C_21_H_21_NO_3_ [M+H]^+^ calculated 336.15942 *m*/*z*, found 336.15980 *m*/*z*.

*N-[3-(But-2-yloxy)phenyl]-3-hydroxynaphthalene-2-carboxamide* (**8b**). Yield 71%; Mp. 152–155 °C; IR (Zn/Se ATR, cm^−^^1^): 2967, 2918, 2873, 1637, 1623, 1590, 1544, 1451, 1267, 1224, 1155, 1133, 1092, 1066, 1005, 915, 881, 869, 847, 765, 748, 709; ^1^H-NMR (DMSO-*d*_6_), δ: 11.29 (s, 1H), 10.54 (s, 1H), 8.48 (s, 1H), 7.93 (d, *J* = 8.1 Hz, 1H), 7.77 (d, *J* = 8.1 Hz, 1H), 7.51 (td, *J* = 7.0 Hz, 1.1 Hz, 1H), 7.47 (s, 1H), 7.36 (td, *J* = 8.1 Hz, 1.1 Hz, 1H), 7.33 (s, 1H), 7.25–7.28 (m, 2H), 6.67–6.73 (m, 1H), 4.36 (sx, *J* = 6.2 Hz, 1H), 1.50–1.80 (m, 2H), 1.25 (d, *J* = 5.9 Hz, 3H), 0.94 (t, *J* = 7.3 Hz, 3H); ^13^C-NMR (DMSO-*d*_6_), δ: 165.63, 158.09, 153.66, 139.67, 135.74, 130.42, 129.57, 128.69, 128.11, 126.87, 125.77, 123.74, 121.97, 112.45, 111.25, 110.53, 107.89, 74.23, 28.56, 19.05, 9.55; HR-MS: for C_21_H_21_NO_3_ [M+H]^+^ calculated 336.15942 *m*/*z*, found 336.15970 *m*/*z*.

*N-[4-(But-2-yloxy)phenyl]-3-hydroxynaphthalene-2-carboxamide* (**8c**). Yield 54%; Mp. 170–172 °C; IR (Zn/Se ATR, cm^−^^1^): 2964, 2926, 2872, 1634, 1602, 1557, 1505, 1495, 1447, 1357, 1345, 1208, 1171, 1123, 1069, 986, 912, 868, 827, 739, 703; ^1^H-NMR (DMSO-*d*_6_), δ: 11.49 (s, 1H), 10.51 (s, 1H), 8.53 (s, 1H), 7.92 (d, *J* = 8.1 Hz, 1H), 7.76 (d, *J* = 8.1 Hz, 1H), 7. 64 (d, *J* = 9.2 Hz, 2H), 7.51 (td, *J* = 7.0 Hz, 1.1 Hz, 1H), 7.36 (td, *J* = 7.0 Hz, 1.1 Hz, 1H), 7.32 (s, 1H), 6.93 (d, *J* = 9.2 Hz, 2H), 4.36 (sx, *J* = 6.0 Hz, 1H), 1.46–1.77 (m, 2H), 1.22 (d, *J* = 6.1 Hz, 3H), 0.93 (t, *J* = 7.3 Hz, 3H); ^13^C-NMR (DMSO-*d*_6_), δ: 165.60, 154.41, 154.18, 135.77, 131.10, 130.08, 128.63, 128.05, 126.75, 125.71, 123.65, 122.35, 120.98, 115.82, 110.57, 74.40, 28.48, 18.99, 9.41; HR-MS: for C_21_H_21_NO_3_ [M+H]^+^ calculated 336.15942 *m*/*z*, found 336.15988 *m*/*z*.

### 3.3. In Vitro Antibacterial Susceptibility Testing

The synthesized compounds were evaluated for *in vitro* antibacterial activity against representatives of multidrug-resistant bacteria, clinical isolates of methicillin-resistant *Staphylococcus aureus* (MRSA) 63718, SA 630 and SA 3202 that were obtained from the National Institute of Public Health (Prague, Czech Republic). *Staphylococcus aureus* ATCC 29213 was used as a reference and quality control strain. Ampicillin (Sigma-Aldrich) was used as the standard. Prior to testing, each strain was passaged onto nutrient agar (Oxoid, Hampshire, UK) with 5% of bovine blood, and bacterial inocula were prepared by suspending a small portion of bacterial colony in sterile phosphate buffered saline (pH 7.2–7.3). The cell density was adjusted to 0.5 McFarland units using a densitometer (Densi‑La‑Meter, LIAP, Riga, Latvia). The final inoculum was made to a 1:20 dilution of the suspension with the Mueller-Hinton broth (MH broth). The compounds were dissolved in DMSO (Sigma), and the final concentration of DMSO in the MH broth (Oxoid) did not exceed 2.5% of the total solution composition. The final concentrations of the evaluated compounds ranging from 256 μg/mL to 0.008 μg/mL. The broth dilution micro-method modified according to NCCLS guidelines [44,45] in MH broth was used to determine the minimum inhibitory concentration (MIC). Drug-free controls, sterility controls and controls consisted of MH broth and DMSO alone were included. The determination of results was performed visually after 24 h of static incubation in the darkness at 37 °C in an aerobic atmosphere. The MICs were defined as the lowest concentration of the compound at which no visible bacterial growth was observed. The results are summarized in Table 1.

### 3.4. In Vitro Antimycobacterial Evaluation

*Mycobacterium tuberculosis* H37Ra ATCC 25177 and well characterised clinical isolate *M.* *avium* subsp. *paratuberculosis* CIT03 were grown in Middlebrook broth (MB), supplemented with Oleic-Albumin-Dextrose-Catalase supplement (Becton Dickinson, Oxford, UK) and mycobactin J (2 μg/mL). Identification of these isolates was performed using biochemical and molecular protocols. At log phase growth, a culture sample (10 mL) was centrifuged at 15,000 rpm/20 min using a bench top centrifuge (Model CR 4-12, Jouan Inc., Winchester, VA, USA). Following removal of the supernatant, the pellet was washed in fresh Middlebrook 7H9GC broth and re-suspended in fresh supplemented MB (10 mL). The turbidity was adjusted to match McFarland standard No. 1 (3 × 10^8^ cfu) with MB broth. A further 1:20 dilution of the culture was then performed in MB broth. The antimicrobial susceptibility of all three mycobacterial species was investigated in a 96-well plate format. In these experiments, sterile deionised water (300 µL) was added to all outer-perimeter wells of the plates to minimize evaporation of the medium in the test wells during incubation. Sample wells were composed of 100 µL of test compound dilution and 100 µL of the bacterial stock being tested against. Dilutions of each compound were prepared in duplicate. For all synthesized compounds, final concentrations ranged from 1000 μg/mL to 8 μg/mL. All compounds were prepared in DMSO and subsequent dilutions were made in supplemented MB. The plates were sealed with parafilm and incubated at 37 °C, for 7 days in the case of *M. tuberculosis* and 11 days in the case of *M. avium* subsp. *paratuberculosis*. Following incubation, a 10% addition of alamarBlue (AbD Serotec, Kidlington, UK) was mixed into each well and readings at 570 nm and 600 nm were taken, initially for background subtraction and subsequently after 24 h re-incubation. The background subtraction is necessary for strongly coloured compounds, where the colour may interfere with the interpretation of any colour change. For non-interfering compounds, a blue colour in the well was interpreted as an absence of growth and a pink colour was scored as growth. The minimum inhibitory concentration (MIC) was defined as the lowest concentration of the compound at which no visible bacterial growth was observed, *i.e.*, the MIC is the lowest concentration that prevented a visual colour change from blue to pink. The MIC value is routinely and widely used in bacterial assays and is a standard detection limit according to the Clinical and Laboratory Standards Institute (CLSI,) [27]. Rifampicin (Sigma-Aldrich) was used as the standard as it is clinically used antimycobacterial drugs. The results are summarized in Table 1.

For the MTT assay, the outer wells of a 96-well plate were filled with 200 µL of sterile water, and the inner wells were filled with 100 µL of the tested compound at the concentration to be examined. Compounds were prepared as previously stated and diluted in Middlebrook media to achieve the desired concentration. *Mycobacterium tuberculosis* H37Ra ATCC 25177 was suspended in ODAC supplemented Middlebrook broth at a MacFarland standard of 1.0 and then diluted through a 1:20, using Middlebrook broth as a diluent. The diluted mycobacteria (100 µL) were added to each well containing the compound to be tested. A negative growth control was composed of 100 µL of DMSO and 100 µL of media, and the diluted mycobacteria in broth absent of inhibiting compounds were used as a positive growth control. All compounds and controls were prepared in triplicate. Plates were incubated at 37 °C for 7 days. After the incubation period, 10% well volume of MTT reagent was mixed into each well and incubated at 37 °C for 24 h. The reagent and media were then aspirated from the wells, to which 50 µL 99% isopropanol was then added, and plates were read at 570 nm. The absorbance readings from the cells, grown in the presence of the tested compounds, were compared with uninhibited cell growth (using DMSO as the blank) to determine the relative percent viability. The percent viability was determined through the MTT assay. The percent viability is calculated through comparison of a measured value against that of the uninhibited control: %viability = OD_570_E/OD_570_P × 100, where OD_570_E is the reading from the compound-exposed cells, while OD_570_P is the reading from the uninhibited cells (positive control). Cytotoxic potential is determined by a percent viability of <70%. 

### 3.5. In Vitro Cytotoxicity Assay

Human monocytic leukemia THP-1 cells were obtained from the European Collection of Cell Cultures (ECACC, Salisbury, UK; Methods of characterization: DNA Fingerprinting (Multilocus probes) and isoenzyme analysis). These cells were routinely cultured in RPMI 1640 (Lonza, Verviers, Belgium) medium supplemented with 10% fetal bovine serum (Sigma-Aldrich), 2% l-glutamine, 1% penicillin and streptomycin (Lonza) at 37 °C with 5% CO_2_. Cells were passaged at approximately 1 week intervals. Cells were routinely tested for the absence of mycoplasma (Hoechst 33258 staining method). The tested compounds were dissolved in DMSO (Sigma-Aldrich) and added in five increasing concentrations to the cell suspension in the culture medium. The maximum concentration of DMSO in the assays never exceeded 0.1%. Subsequently, the cells were incubated for 24 h at 37 °C with 5% CO_2_ to various compound concentrations ranging from 0.37 to 20 μM in RPMI 1640 medium. Cell toxicity was determined using a Cytotoxicity Detection Kit^PLUS^ Lactate dehydrogenase (LDH) assay kit (Roche Diagnostics, Mannheim, Germany) according to the manufacturer’s instructions, as described previously [17,18,46]. For LDH assays, cells were seeded into 96-well plates (5 × 10^4^ cells·well^−1^ in 100 μL culture medium) in triplicate in serum-free RPMI 1640 medium, and measurements at 492 nm wavelength (Synergy 2 Multi-Mode Microplate Reader, BioTek, Winooski, VT, USA) were taken 24 h after the treatment with tested compounds. The median lethal dose values, LD_50_, were deduced through the production of a dose-response curve. All data were evaluated using GraphPad Prism 5.00 software (GraphPad Software, San Diego, CA, USA). The results are summarized in Table 1.

### 3.6. Study of Inhibition Photosynthetic Electron Transport (PET) in Spinach Chloroplasts

Chloroplasts were prepared from spinach (*Spinacia oleracea* L.) according to Masarovicova and Kralova [47]. The inhibition of photosynthetic electron transport (PET) in spinach chloroplasts was determined spectrophotometrically (Genesys 6, Thermo Scientific Corporation), using an artificial electron acceptor 2,6-dichlorophenol-indophenol (DCPIP) according to Kralova *et al.* [48], and the rate of photosynthetic electron transport was monitored as a photoreduction of DCPIP. The measurements were carried out in phosphate buffer (20.0 mM, pH 7.2) containing sucrose (0.4 M), MgCl_2_ (5.0 mM) and NaCl (15.0 mM). The chlorophyll content was 30 mg/L in these experiments and the samples were irradiated (~100 W/m^2^ with 10 cm distance) with a halogen lamp (250 W) using a 4 cm water filter to prevent warming of the samples (suspension temperature 22 °C). The studied compounds were dissolved in DMSO due to their limited aqueous solubility. The applied DMSO concentration (up to 4%) had negligible effect on the photochemical activity in isolated spinach chloroplasts (observed differences in DCPIP photoreduction due DMSO addition were within experimental error). The inhibitory efficiency of the studied compounds was expressed by IC_50_ values, *i.e.*, by molar concentration of the compounds causing 50% decrease in DCPIP photoreduction relative to the untreated control. The comparable IC_50_ value for a selective herbicide 3-(3,4-dichlorophenyl)-1,1-dimethylurea, DCMU (Diuron^®^) was about 1.9 μM. All reagents used in this study were of analytical grade and were purchased from Sigma. The results are summarized in Table 1.

The emission fluorescence spectra were recorded on a fluorescence spectrophotometer F-2000 (Hitachi, Tokyo, Japan) at room temperature (24 °C). The samples of chloroplast suspension (10 mg chlorophyll/L) with and without the studied inhibitor were excited at 436 nm, using an excitation slit 20 nm and emission slit 10 nm, and were kept in the dark for 2 min prior to the measurement. Due to low aqueous solubility the compounds were added to a chloroplast suspension in DMSO solution. The DMSO concentration in all samples was the same as in the control (10%).

## 4. Conclusions

A series of fifteen new *N*-alkoxyphenylanilides of 3-hydroxynaphthalene-2-carboxylic acid was prepared by means of microwave synthesis and subsequently characterized. All compounds were tested for their *in vitro* antimicrobial activity against *S. aureus*, three methicillin-resistant *S. aureus* strains, *M. tuberculosis* H37Ra and clinical isolate of *M. avium* subsp. *paratuberculosis* as well as for their ability to inhibit photosynthetic electron transport (PET) in spinach chloroplasts (*Spinacia oleracea* L.)*.* 3-Hydroxy-*N*-(2-propoxyphenyl)naphthalene-2-carboxamide (**5a**) and *N*-[2-(but-2-yloxy)phenyl]-3-hydroxynaphthalene-2-carboxamide (**8a**) showed MIC = 12 µM against all methicillin-resistant *S. aureus* strains, 4-fold higher than that of ampicillin. Compound **8a** and 3-hydroxy-*N*-[3-(prop-2-yloxy)phenyl]naphthalene-2-carboxamide (**7b**) showed MICs = 23 µM and 24 µM against *M. tuberculosis* respectively; **8a** also demonstrated the highest activity (MIC = 89 µM) against *M. avium* subsp. *paratuberculosis*.Compound **8a** was the most potent antimicrobial compound within the whole series. Based on the results obtained using standard MTT assay it may be hypothesized that the mechanism of action of the studied compounds could be connected with an effect on mycobacterial energy metabolism. Screening of cytotoxicity performed using the THP-1 cells proved no significant lethal effect of the discussed compounds except for *N*-[3-(but-2-yloxy)phenyl]-3-hydroxynaphthalene-2-carboxamide (**8b**) that demonstrated fairly high toxicity LD_50_ = 2.7 ± 0.7 µM. *N*-(3-Ethoxyphenyl)-3-hydroxynaphthalene-2-carboxamide (IC_50_ = 4.5 µM) was the most active PET inhibitor. The compounds caused perturbation of Chl*a-*protein complexes in the thylakoid membrane, and the section at Q_B_ site on the acceptor side of PS II was estimated as inhibitory site of action of the studied compounds. Biological activity of the compounds is dependent on substitution of Cʹ_(2)_ or Cʹ_(3)_ position by the alkoxy chain and its bulkiness. Antimicrobial activity and cytotoxicity (strongly related to Cʹ_(3)_ substitution) is connected especially with the elongation of the alkoxy tail and increasing surface activity of the compounds, while PET inhibition (strongly related to Cʹ_(3)_ substitution) is connected with decreasing surface activity and a rather short and unbranched alkoxy chain. The effect of the additional prolongation and/or branching of the alkoxy tail as well as the change of the position of the phenolic moiety on the naphthalene scaffold has been under investigation.
